# Prediction of reversible disulfide based on features from local structural signatures

**DOI:** 10.1186/s12864-017-3668-8

**Published:** 2017-04-04

**Authors:** Ming-an Sun, Yejun Wang, Qing Zhang, Yiji Xia, Wei Ge, Dianjing Guo

**Affiliations:** 1 School of Life Sciences and the State Key Laboratory of Agrobiotechnology, The Chinese University of Hong Kong, Shatin, New Territories, Hong Kong, SAR China; 2 Department of Cell Biology and Genetics, School of Basic Medical Sciences, Shenzhen University Health Science Center, Nanhai Ave 3688, Shenzhen, 518060 China; 3 Department of Biology, Hong Kong Baptist University, Kowloon Tong, Kowloon, Hong Kong, SAR China; 4 Centre of Reproduction, Development and Aging, Faculty of Health Sciences, University of Macau, Taipa, Macau, China

**Keywords:** Reversible disulfide, Structural disulfide, Cysteine, Structural signature, Redox, SVM, Prediction, RevssPred

## Abstract

**Background:**

Disulfide bonds are traditionally considered to play only structural roles. In recent years, increasing evidence suggests that the disulfide proteome is made up of structural disulfides and reversible disulfides. Unlike structural disulfides, reversible disulfides are usually of important functional roles and may serve as redox switches. Interestingly, only specific disulfide bonds are reversible while others are not. However, whether reversible disulfides can be predicted based on structural information remains largely unknown.

**Methods:**

In this study﻿, two datasets with both types of disulfides were compiled using independent approaches. By comparison of various features extracted from the local structural signatures, we identified several features that differ significantly between reversible and structural disulfides, including disulfide bond length, along with the number, amino acid composition, secondary structure and physical-chemical properties of surrounding amino acids. A SVM-based classifier was developed for predicting reversible disulfides.﻿

**Results:**

By 10-fold cross-validation, the model achieved accuracy of 0.750, sensitivity of 0.352, specificity of 0.953, MCC of 0.405 and AUC of 0.751 using the RevSS_PDB dataset. The robustness was further validated by using RevSS_RedoxDB as independent testing dataset. This model was applied to proteins with known structures in the PDB database. The results show that one third of the predicted reversible disulfide containing proteins are well-known redox enzymes, while the remaining are non-enzyme proteins. Given that reversible disulfides are frequently reported from functionally important non-enzyme proteins such as transcription factors, the predictions may provide valuable candidates of novel reversible disulfides for further experimental investigation.

**Conclusions:**

This study provides the first comparative analysis between the reversible and the structural disulfides. Distinct features remarkably different between these two groups of disulfides were identified, and a SVM-based classifier for predicting reversible disulfides was developed accordingly. A web server named RevssPred can be accessed freely from: http://biocomputer.bio.cuhk.edu.hk/RevssPred.

**Electronic supplementary material:**

The online version of this article (doi:10.1186/s12864-017-3668-8) contains supplementary material, which is available to authorized users.

## Background

Disulfide bonds are formed between the sulfur atoms of pairs of cysteine residues within or across proteins. With the exception of peptide bonds, disulfide bonds are the most common covalent linkages of amino acids in proteins. For example, about 10% of proteins made by mammalian cells contain disulfide bonds [1]. Disulfide bonds play critical roles in protein stability and function, and they are generally considered to be highly conserved during evolution. Moreover, the rate of disulfide bond acquisition shows a strong positive correlation with the complexity of living organism [2].

Disulfide bonds have been generally considered as of pure structural roles. Those structural disulfides can help stabilize the tertiary or quaternary structure of proteins, and they cannot be easily opened once formed [3]. Later on, it was found that some disulfides may contribute little to protein stabilization, and others may actually destabilize their resident protein [4, 5]. Interestingly, some disulfide bonds can even be reversibly oxidized and reduced under different conditions. Thus, the current view is that disulfide proteome consists of two sub-groups: a structural group and a reversible (redox-sensitive) group [6].

Unlike structural disulfides which are inert and inactive, reversible disulfides are usually of important functional roles, and may serve as redox switches [3, 7–9]. One well studied example is the *Escherichia coli* OxyR transcription factor, which senses the H_2_O_2_ and can be activated through the formation of an intramolecular disulfide bond [10, 11]. Some reversible disulfides, such as those at the active sites of well studied thiol-disulfide oxidoreductases, are of catalytic function. Other reversible disulfides may also control protein function by triggering a conformational change when formed or broken [1]. According to previous studies, formation of reversible disulfide seems to be one major type of protein cysteine oxidative modification [3, 12]. Due to their functional importance, reversible disulfides have caught much attention in the past decade [5, 8, 9, 13–16].

A few studies analyzed the redox potentials of the active disulfides in protein disulfide isomerase (DsbA) and thioredoxin [17–19], which are well known redox proteins. One other study attempted to detect the catalytic redox-active cysteine residues from thiol oxidoreductases [20]. However, these studies were focused only on reversible disulfides in specific types of well-known redox enzymes such as oxidoreductases, and utilized rather small datasets for analysis. Nevertheless, it has been reported that many reversible disulfides were also detected from functionally important non-enzyme proteins such as transcription factors [10, 21–23]. The study of reversible disulfides in these non-enzyme proteins may be of particular importance and yet more challenging. Until now, the determinants of the redox potential of disulfides are still poorly understood.

Although computational models have been developed for the prediction of structural disulfides [24–29] and various types of cysteine redox modifications such as S-sulfenylation [30], S-nitrosylation [31–35] and S-glutathionylation [36, 37], to our knowledge, there is still no study focusing on direct comparative analysis and *in silico* prediction of structural and reversible disulfides. So far, the most relevant computational work about reversible disulfide is carried out by Sanchez et al. [38], who analyzed twelve structural features and identified three features useful for the prediction of redox-sensitive cysteines. The three features are: distance to the nearest cysteine sulfur atom, solvent accessibility and pKa. Using these features, they trained a decision-tree based classifier for predicting redox-susceptible cysteines. However, that study is designed for general analysis of various reversibly oxidized cysteines, and no particular analysis was conducted for reversible disulfides. Furthermore, probably due to the limited amount of experimentally verified redox susceptible cysteines, the dataset used by Sanchez et al. [38] is rather small and biased towards several protein families in particular oxidoreductases. Thus, until now the differences between reversible and structural disulfides have never been comprehensively investigated.With the accumulation of known reversible disulfides, comparative analysis between reversible and structural disulfide is highly desirable because it has promising potential in revealing distinct characteristics for reversible disulfides, some of which may be useful for *in silico* prediction of reversible disulfide.

In this study, we compiled two independent datasets with both types of disulfides (named RevSS_PDB and RevSS_RedoxDB) from independent sources, respectively. After extensive analysis of various properties for the disulfide-bonded cysteines and the surrounding structural microenvironment, several remarkable features that differ significantly between reversible and structural disulfides were identified. We demonstrated that these features are efficient for reversible disulfides prediction. A SVM-based classifier named RevssPred were further developed for *in silico* prediction of reversible disulfides from protein structure obtained from the PDB database.

## Methods

### Generation of RevSS_PDB and RevSS_RedoxDB datasets

To generate the RevSS_PDB dataset, 46,944 X-ray crystal structures with resolution better than 2.0 Å were retrieved from the PDB database [39] on December 1, 2015 (Additional file 1: Figure S1). PDB files were first assigned to each species, then those for the same protein were grouped together according to the annotation from the Uniprot database [40]. After excluding proteins with less than two PDB files, we obtained 6,414 proteins with 35,290 PDB files in total. For one protein, each Cys-Cys pair was scanned among different structures. Those form disulfide (intra- or inter-chain disulfide bonds, as indicated by SSBOND parameter) in certain structures and meanwhile remain reduced in other structures were annotated as reversible disulfides. Accordingly, others forming disulfide in all the structures were defined as structural disulfide. When multiple structures were found associated with the same disulfide, only the one with the best resolution were kept for further analysis. After removing redundant proteins with more than 30% similarity using BlastClust [41], followed by manual curation to remove those with similar flanking sequences (10 residues at both sides), the RevSS_PDB dataset consists of 230 reversible disulfides and 450 structural disulfides (Additional file 2: Table S1).

To generate RevSS_RedoxDB dataset, we first retrieved 235 known reversible disulfides from the RedoxDB [42] which is a manually curated database for various types of experimentally validated redox-sensitve cysteines. We made further screening for each reversible disulfide to ascertain that at least one associated X-ray crystal structure with resolution better than 3.0 Å are available in PDB database. The structure with the best resolution was used when multiple sources of structural data are available for the same disulfide. After removing redundant data with more than 30% similarity by using BlastClust, finally we obtained fifty reversible disulfides (Additional file 3: Table S2). To generate a control dataset with putative structural disulfides, we first retrieved all the X-ray crystal structures with resolution better than 3.0 Å (as annotated by SSBOND parameter) from the PDB database. After excluding PDB files with sequences of more than 30% similarity to the RedoxDB , followed by redundant sequences removal (>30% similarity) using BlastClust, finally we obtained a non-redundant control dataset containing 3,016 putative structural disulfides for comparative analysis.

### Electrostatic properties of disulfide-bonded Cys

Cysteine thiol pKa values were calculated using PROPKA [43]. Since most disulfide-bonded Cys are not ionizable before reduction, PROPKA assigns a disulfide-bonded Cys (with S-S distance <2.5 Å) with a trivial values of 99.99 without further calculation. However, for Cys located in the CXXC motifs, PROPKA always calculates their pKa values even when they are oxidized (bonded). Because we aim to compare the pKa values for the reversible-SS Cys and the structural-SS Cys, we let PROPKA calculate the pKa values for all the cysteines. The Naccess software program (Version 2.1.1) [44] with default settings was used to calculate the cysteine solvent accessibility with the PDB data.

### Structural signatures surrounding disulfides

Based on the coordinates of each amino acid as annotated in the PDB files, the structural signature centered on each cysteine residues for a disulfide was extracted using home-made PERL scripts (Additional file 4: Figure S2). The default radius of 10 Å was used according to previous studies [45], and the two disulfide-bonded cysteines were excluded from the structural signature.

#### Secondary structure of structural signature

Secondary structures for each protein were calculated using STRIDE [46] with default settings. Different types of secondary structure are represented as single letters ("B" for bridge, "C" for coil, "E" for strand, G for 310-helix, "H" for α-helix, "I" for pi-helix and "T" for turn). The predicted secondary structure for each amino acid for the structural signatures was then extracted.

#### Physical-chemical property

Four types of physical-chemical properties of amino acids were considered, including hydrophobicity [47], net charge index of side chains of amino acids (NCI) [48], propensity and side chain pKa value. The physical-chemical property values for each amino acid can be found in Additional file 2: Table S1.

### Support vector machines implementation and parameter optimization

Support vector machine (SVM) [49] is a widely used machine-learning method based on statistical learning theory. It has been successfully applied in many aspects of bioinformatics studies. In this work, SVM technique was implemented using LIBSVM 3.12 [50]. The radial basis function (RBF kernel) is used, which is defined as:

$$ K\left({x}_i, x\right)= \exp \left(-\gamma \left\Vert {x}_i- x\right\Vert \right) $$

where *x* and *x*_*i*_ are two data vectors and *γ* is a training parameter. The regularization parameter C and the kernel parameter *γ* were optimized by a grid search approach using 10-fold cross-validation.

### Performance assessment

The model’s performance is evaluated using various criteria including sensitivity (SN), specificity (SP), accuracy (ACC) and Matthews correlation coefficient (MCC). They are defined as below:

$$ \begin{array}{l} SN=\frac{TP}{TP+ FN}\hfill \\ {} SP=\frac{TN}{TN+ FP}\hfill \\ {} ACC=\frac{TP+ TN}{TP+ TN+ FP+ FN}\hfill \\ {} MCC=\frac{\left( TP\times TN\right)+\left( FP\times FN\right)}{\sqrt{\left( TP+ FP\right)\times \left( TP+ FN\right)\times \left( TN+ FP\right)\times \left( TN+ FN\right)}}\hfill \end{array} $$

where TP, TN, FP, and FN denotes the numbers of true positives, true negatives, false positives, and false negatives, respectively.

The model’s performance was evaluated using 10-fold cross-validation. The receiver operating characteristic (ROC) curve, which is one of the most robust approaches for classifier evaluation, was obtained by plotting true positive rate (sensitivity) against the false positive rate (1-specificity). The area under the ROC curve (AUC) was also calculated.

### Web server implementation

The web server named RevssPred is implemented using Perl, PHP, MySQL and Apache. To speed up the prediction process, we have pre-computed the result for X-ray crystal structures with resolution better than 2.5 Å from the PDB database. According to the PDB_IDs provided by users, the server will first try to retrieve pre-computed results from the MySQL database. If failed, it then downloads the structural files from the PDB database automatically, extract the required features, and perform *de novo* prediction.

## Results

### Generation of RevSS_PDB and RevSS_RedoxDB datasets

Two datasets, named RevSS_PDB and RevSS_RedoxDB, were generated using independent approaches, respectively. Inspired by one previous study [51], the RevSS_PDB dataset, which consists of 230 reversible disulfides and 450 putative structural disulfides (Additional file 2: Table S1), was generated by detecting disulfides with alternative redox states among different proteins structures (Additional file 4: Figure S2). However, RevSS_RedoxDB dataset which contains 50 reversible disulfides and 3,016 putative structural disulfides (Additional file 5: Table S3), was constructed from RedoxDB database [42]. These two datasets were generated using different procedure and are highly independent from each other, with only six redox proteins commonly exist in both datasets. Further inspection showed that even though both datasets include a large number of well known redox proteins such as oxidoreductases and hydrolases, their protein family compositions are remarkably different (Additional file 6: Figure S3). Both datasets were used for comparative analysis.

### Reversible disulfides are of longer S-S distance

**Fig. 1:**
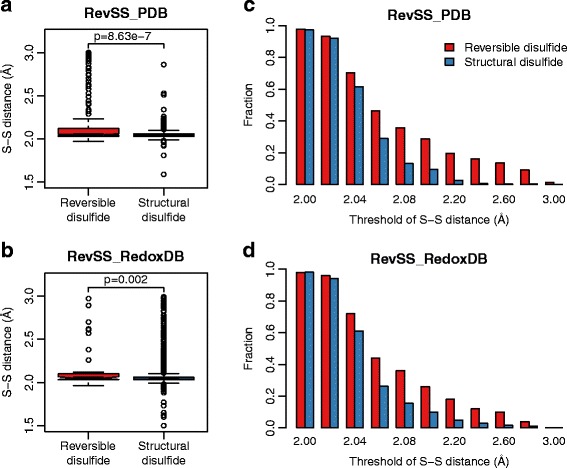
Reversible disulfides have longer S-S distance compared with structural disulfides. This figure shows the comparison of S-S distance between reversible and structural disulfides. **a**, **b** Box-plots showing reversible disulfides have relatively longer S-S distance. **c**, **d** Fraction of disulfides above each specified threshold for reversible and structural disulfides. Result from both RevSS_PDB and RevSS_RedoxDB were demonstrated. P-values from Wilcoxon rank sum test were indicated

As the disulfide-bonded Cys from these two groups of disulfides are with different reversible potential, it is possible that they may have different properties. Thus, we first focused the analysis on the pairs of disulfide-bonded Cys. One important property of a disulfide is the disulfide bond length, which is denoted as the distance between the two thiol groups (S-S distance). Disulfide bonds are usually about 2.05 Å in length, and 3.0 Å is taken as the cutoff for disulfides in the PDB database. We extracted the S-S distance as annotated by the SSBOND parameters in the RevSS_PDB dataset, and made comparison between the reversible and the structural disulfides. To our surprise, reversible disulfides seem to be significantly longer (p = 8.6e-7, Wilcoxon rank sum test) than structural disulfides (Fig. 1a). The average S-S distance was calculated as 2.18 Å for reversible disulfides, and 2.05 Å for structural ones. Using thresholds ranging from 2.0 Å to 3.0 Å, we calculated the proportion of both groups of disulfides above the threshold. Compared with structural disulfides, much higher proportion of reversible disulfides are with longer S-S distance. For example, 28.7% of reversible disulfides are of S-S distance > =2.10 Å. In contrast, only 9.3% of structural disulfides are longer than 2.10 Å (Fig. 1b). The same observation was found when using the RevSS_RedoxDB dataset (Fig. 1c,d), indicating this is likely a general difference between these two groups of disulfides. We further analyzed the electrostatic characteristics of the disulfide-bonded cysteines. However, no significant difference was found between reversible and structural disulfides in terms of acid dissociation constant (pKa) and solvent accessibility (Additional file 7: Figure S4).

To our knowledge, this is the first time showing the remarkable difference of S-S distance between reversible and structural disulfides. It is possible that the relatively longer S-S distance is a unique characteristic for reversible disulfides. The other possibility is that maybe some of those reversible disulfides are under the intermediate state between oxidized and reduced form, given the fact that reversible disulfides can be reduced back under certain condition. The underlying mechanisms for this observation still need further investigation.

### General characteristics of structural signatures

We hypothesized that the surrounding structural microenvironment, in addition to the properties of disulfide-bonded Cys, may also contribute remarkably to the redox potential of reversible disulfides. Some previous studies have linked the spatial microenviroment to the active sites in proteins [45] and cysteines modifiable to cysteine sulfinic acid [52]. Inspired by these studies, we adopted a similar strategy to extract the structural signatures (defined as the amino acids occur within 10 Å from the two disulfide-bonded Cys. See Methods section) for reversible and structural disulfides (Additional file 4: Figure S2), and conducted comparison regarding their amino acid composition, secondary structure and physical-chemical properties.

**Fig. 2:**
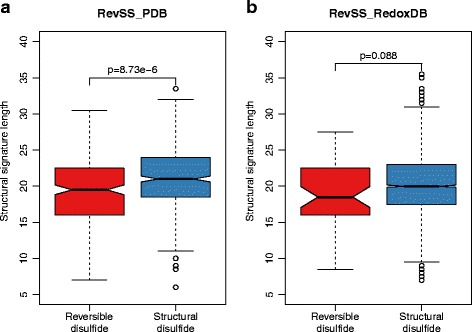
Difference in structural signature length between structural and reversible disulfides. Results from both RevSS_PDB (**a**) and RevSS_RedoxDB (**b**) were demonstrated. P-values from Two-tailed Student’s t-test were indicated

Initially, we asked if the length of the structural signature (which represents the number of amino acids surrounding the disulfide) is different between the two types of disulfides. Using the RevSS_PDB dataset, we found that the structural signatures are significantly shorter in reversible disulfides (Fig. 2a; p = 8.7e-6 by Two-tailed Student’s t-test). The same result was obtained when the RevSS_RedoxDB dataset was tested (Fig. 2b). This implies that the densities of the surrounding amino acids may affect the reversible potential of a disulfide.

### Amino acid composition and secondary structure of the structural signatures

We also examined the amino acid composition of structural signatures, and found that several amino acids show different frequency surrounding reversible and structural disulfides. The disulfide-bonded Cys were excluded from further analysis. From the RevSS_PDB dataset, we found that cysteines are significantly under-represented whereas serines were over-represented surrounding reversible disulfides (Additional file 8: Figure S5; Bonferroni-adjusted p = 0.036, Two-tailed Student’s t-test). The RevSS_RedoxDB dataset shows similar result (Fig. 5b), with the exception that phenylalanines are over-represented instead.

**Fig. 3:**
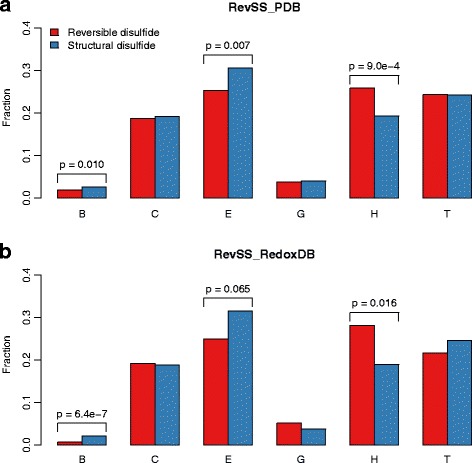
Secondary structure compositions for structural signatures of reversible and structural disulfides. x-axis denotes different types of secondary structure. y-axis gives the frequency of each types of secondary structure in the structural signatures. Result from both RevSS_PDB (**a**) and RevSS_RedoxDB (**b**) were demonstrated. P-values determined by Two-tailed Student's t-test were indicated

We further examined the secondary structure for residues involved in the structural signatures. Most of these residues for both types of disulfide-bonded Cys form coil, strand, α-helix and turn. The frequencies for three types of secondary structure, including bridge, strand and α-helix, are remarkably different between reversible and structural disulfides in both datasets (Fig. 3). Among them, bridge and strand are over-represented surrounding reversible disulfides, while α-helix is under-represented. This result is in accordance with one previous research, which reported a marked preference for α-helix and disfavor of β-strand around the redox-active Cys in thiol oxidoreductases [20].

### Physical-chemical properties of structural signatures

**Fig. 4:**
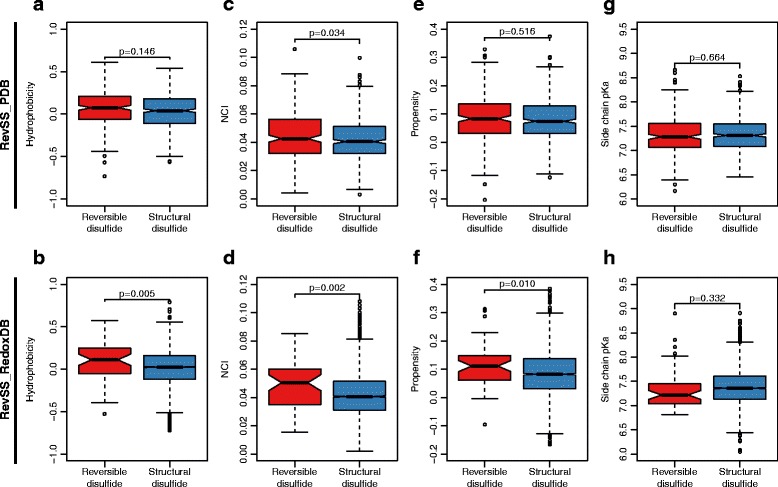
Comparison of mean values for physical-chemical property of amino acids in the structural signatures. This figure shows the comparison of hydrophoticity (**a**, **b**), NCI (**c**, **d**), propensity (**e**, **f**) and side chain pKa (**g**, **h**) between reversible and structural disulfides. Results from both RevSS_PDB and RevSS_RedoxDB were demonstrated. P-values from Two-tailed Student's t-tests were indicated

To understand how physical-chemical properties of the surrounding amino acids may affect the reversible potential of a disulfide, we further examined the hydrophobicity, net charged index (NCI), propensity and side chain pKa for the extracted structural signatures. We found that the values for three of the properties (hydrophobicity, NCI and propensity) are significantly higher for structural signatures from reversible disulfides (Fig. 4a-f). However, the side chain pKa shows no significant difference between the structural signature of these two types of disulfides (Fig. 4g,h).

### Prediction of reversible disulfide using identified features

We identified several features that are significantly different between reversible and structural disulfides, including the S-S distance (DST), along with the length (LEN), amino acid composition (AAC), secondary structure (SST) and physical-chemical properties (PCP) of the structural signatures. It is desirable to further examine if these features can be used for *in silico* prediction of reversible disulfides.

**Fig. 5:**
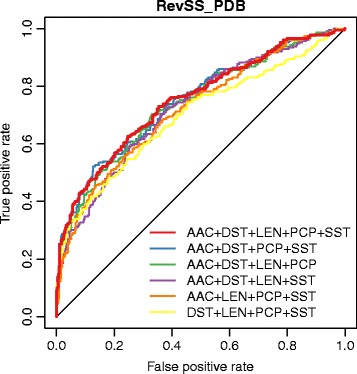
The ROC curves based on 10-fold cross-validation with different combinations of features using RevSS_PDB dataset

Various combinations of these features were used to train different SVM models. The performance was first evaluated by 10-fold cross-validation using RevSS_PDB dataset (Table 1; Fig. 5). When all of these features were utilized, the model achieved accuracy of 0.750, sensitivity of 0.352, specificity of 0.953, MCC of 0.405 and AUC of 0.751 (Table 1). Removing any of these features will reduce the AUC value and also negatively affect other parameters. Thus, the combination of AAC + DST + LEN + PCP + SST (as 32-dimentional vector) was selected as the final optimized feature set. By grid search using 10-fold cross-validation, the regularization parameter C and the kernel parameter *γ* for SVM classifier were optimized as 32768.0 and 0.00048828125, respectively.

**Table 1:** Performance evaluation for different combinations of features by 10-fold cross-validation using RevSS_PDB dataset

Feature sets	ACC	SN	SP	MCC	AUC
AAC + DST + LEN + PCP + SST	0.750	0.352	0.953	0.405	0.751
AAC + DST + PCP + SST	0.743	0.339	0.949	0.383	0.749
AAC + DST + LEN + PCP	0.738	0.291	0.967	0.375	0.740
AAC + DST + LEN + SST	0.728	0.283	0.956	0.341	0.726
AAC + LEN + PCP + SST	0.729	0.339	0.929	0.344	0.719
DST + LEN + PCP + SST	0.729	0.265	0.967	0.348	0.698

The results were ordered by AUC values

When further evaluated by 10-fold cross-validation using a balanced RevSS_RedoxDB (structural disulfides were randomly down-sampled to 100) dataset, similar result with accuracy of 0.755, sensitivity of 0.560, specificity of 0.852, MCC of 0.429 and AUC of 0.780 was obtained (Additional file 9: Figure S6). To further validate the robustness of the method, we applied the SVM-classifier trained using the RevSS_PDB dataset to the non-redundant balanced RevSS_RedoxDB dataset (see Methods section). The accuracy, sensitivity, specificity, and MCC reach 0.726, 0.250, 0.964 and 0.327 respectively. Taken together, these results indicate that the features identified in this study are efficient and robust for predicting reversible disulfides.

### Application and web server implement

A SVM-classifier named RevssPred was trained with the AAC + DST + LEN + PCP + SST feature set using the RevSS_PDB dataset, and applied to all the X-ray crystal structures with resolution better than 2.5 Å (around 20,000 in total) in the PDB database. Among these protein structures, 7.8% (6,792 out of 87,608) of disulfides are predicted as putative reversible disulfides. A careful examination of the 97 non-redundant (with less than 30% similarity to each other; sequences with >30% similarity to any of RevSS_PDB sequences discarded) representative human proteins with predicted reversible disulfide revealed that 32.0% (31 out of 97) of them are well studied redox proteins such as hydrolases, transferases and oxidoreductases, while the remaining 68.0% are non-enzyme proteins (Additional file 10: Table S4, Additional file 11: Figure S7). The percentage of non-enzyme proteins is two times higher than that predicted from the Revss_PDB dataset (68.0% vs. 36.0%), probably due to the fact that previous studies are biased toward several families of redox enzymes. Given that reversible disulfides are frequently reported from functionally important non-enzyme proteins such as transcription factors, the prediction may provide valuable candidates of novel reversible disulfides for further experimental analysis.

These pre-computed results, all the datasets used in this study, together with the SVM classifier trained as abovementioned, were incorporated in a web server named RevssPred which can be accessed freely. The web server takes a list of PDB_IDs as input. It first tries to retrieve pre-computed result from the MySQL database. If failed, it will then download the structural files from the PDB database automatically and initiate the *de novo* prediction.

## Discussion

In this study, an extensive comparison for features extracted from local structural signatures was made between reversible and structural disulfides. Several features were found to be remarkably different between these two groups, including the S-S distance, along with the length, amino acid composition, secondary structure and physical-chemical properties of the structural signatures. By combining these feature sets, we further developed RevssPred which is efficient for the prediction of reversible disulfides. Many of these distinctive features identified in this study were reported for the first time. Our results indicate that the local structural microenvironment is of vital importance for determining the reversible potential of disulfide.

To date, reversible disulfides have mostly been studied in well-known redox enzymes such as thiol oxidoreductases. However, reversible disulfide could also serve as redox switches for many non-enzyme proteins such as transcription factors [53]. The reversible disulfides in these non-enzyme proteins may be of particular functional importance. Unlike previous studies which only focused on specific types of well-known redox enzymes, our study covered the reversible disulfides from a broad range of proteins. Interestingly, when RevssPred was applied to known structures in the PDB database, more than half of the predicted reversible disulfide containing proteins are non-enzyme proteins. The prediction may provide valuable candidates of novel reversible disulfides for further experimental analysis.

As the first comparative and predictive analyses made directly between reversible and structural disulfide, our study also has its limitations. Firstly, even though we tried to compile the most extensive training datasets using two independent approaches from all available resources, the two datasets utilized here are still relatively small. This is due to the difficulties in experimental identification of reversible disulfides and the fact that the protein structures are only available for a small fraction of proteins in the proteome. Secondly, the method proposed in this study requires structure data from the PDB database, which is not available for many proteins. Even so, we have illustrated its feasibility and efficiency in accurate prediction of reversible disulfides. A valuable list of putative reversible disulfides were provided to the research community for further experimental validation. With the accumulation of data for verified reversible disulfides, we expect that predictive models based on sequences or predicted structures will find wide application in the future.

## Conclusions

To the best of our knowledge, this study represents by far the most extensive comparison made between reversible and structural disulfides. It is also the first attempt in *de novo* prediction of reversible disulfides. This study not only opens the possibility of deriving mechanistic insights into the determinants of disulfide redox potential, but also guides further experimental discovery and validation of reversible disulfides.

## Additional files

Additional file 1: Figure S1. Workflow for the generation of RevSS_PDB dataset by parsing protein structures from PDB database (PDF). RevSS_PDB dataset was generated from X-ray crystal structures of resolution better than 2.0 Å from PDB database. In brief, PDB files from the same proteins of the same species were first grouped together. For each group of PDB files, each Cys-Cys pair was scanned among different structures, and those represent as disulfide and reduced state in different structures were annotated as reversible disulfides. After redundancy removal using BlastClust followed by manual curation, the final RevSS_PDB dataset which contains both structural and reversible disulfides was generated. (PDF 78 kb)

Additional file 2: Table S1. Physical-chemical properties of amino acids (XLS). (XLSX 9 kb)

Additional file 3: Table S2. The detailed information for RevSS_PDB (XLS). (XLSX 36 kb)

Additional file 4: Figure S2. Extraction of structural signatures surrounding disulfide in the protein structure (PDF). (a) The three dimensional structure of OxyR (PDB_ID is 1L6A) which is a transcription factor important for oxidative stress response in bacteria. The disulfide bond formed between C199 and C208 is shown as a pink bar. (b) For each Cys, the surrounding segments (within 10Å to disulfide-bonded Cys) are extracted and then combined into the so-called “structural signatures” according to their primary sequence, respectively. The signatures from the two disulfide-bonded Cys are then merged together for analysis. (PDF 374 kb)

Additional file 5: Table S3. The detailed information for RevSS_RedoxDB (XLS). (XLSX 129 kb)

Additional file 6: Figure S3. Enzyme classification of reversible disulfide containing proteins in the training datasets (PDF). This figure was modified from the “Enzyme Classification” result obtained from PDB database. Only reprehensive proteins of less than 30% similarity to each other are used to generate this figure. Proteins not assigned to known enzyme groups were labeled as “Unknown”. (PDF 46 kb)

Additional file 7: Figure S4. Comparison of the pKa and solvent accessibility between reversible and structural disulfides (PDF). This figure shows the comparison of pKa (a,b) and solvent accessibility (c,d) between reversible and structural disulfides. Results from RevSS_PDB and RevSS_RedoxDB were both demonstrated. P-values from Two-tailed Student's t-test were indicated. (PDF 136 kb)

Additional file 8: Figure S5. Amino acid composition of the structural signatures for reversible and structural disulfides (PDF). x-axis denotes amino acid types, and y-axis gives the fraction of each amino acid in the structural signatures. Statistical significance was determined by Two-tailed Student's t-test, and Bonferroni-adjusted p-values were denoted by * when *p* < 0.05, ** when *p* < 0.01, and *** when *p* < 0.001. (PDF 101 kb)

Additional file 9: Figure S6. ROC curve based on 10-fold cross-validation for RevSS_RedoxDB dataset (PDF). The AUC values and feature sets were indicated. (PDF 77 kb)

Additional file 10: Table S4. Representative human proteins with predicted reversible disulfides (XLS). (XLSX 36 kb)

Additional file 11: Figures S7. Enzyme classification of proteins with predicted reversible disulfides in human (PDF). This figure was modified from the “Enzyme Classification” result obtained from PDB database. Only reprehensive proteins of less than 30% similarity to each other, and of less than 30% similarity to any of the RevssPDB sequences were used for generating this figure. Proteins not assigned to known enzyme groups were labeled as “Unknown”. (PDF 43 kb)

## Abbreviations

AAC: Amino acid composition
ACC: Accuracy
AUC: Area under the ROC curve
DST: S-S distance
LEN: Length
MCC: Matthews correlation coefficient
PCP: Physical-chemical property
ROC: Receiver operating characteristic
SN: Sensitivity
SP: Specificity
SST: Secondary structure
SVM: Support vector machine
