# Supplementary figures and images for: Prediction of reversible disulfide based on features from local structural signatures
Source: BMC Genomics. 2017 Apr 4;18:279. doi: 10.1186/s12864-017-3668-8 (PMC5379614; doi:10.1186/s12864-017-3668-8)

# Extract reversible disulfide from PDB database

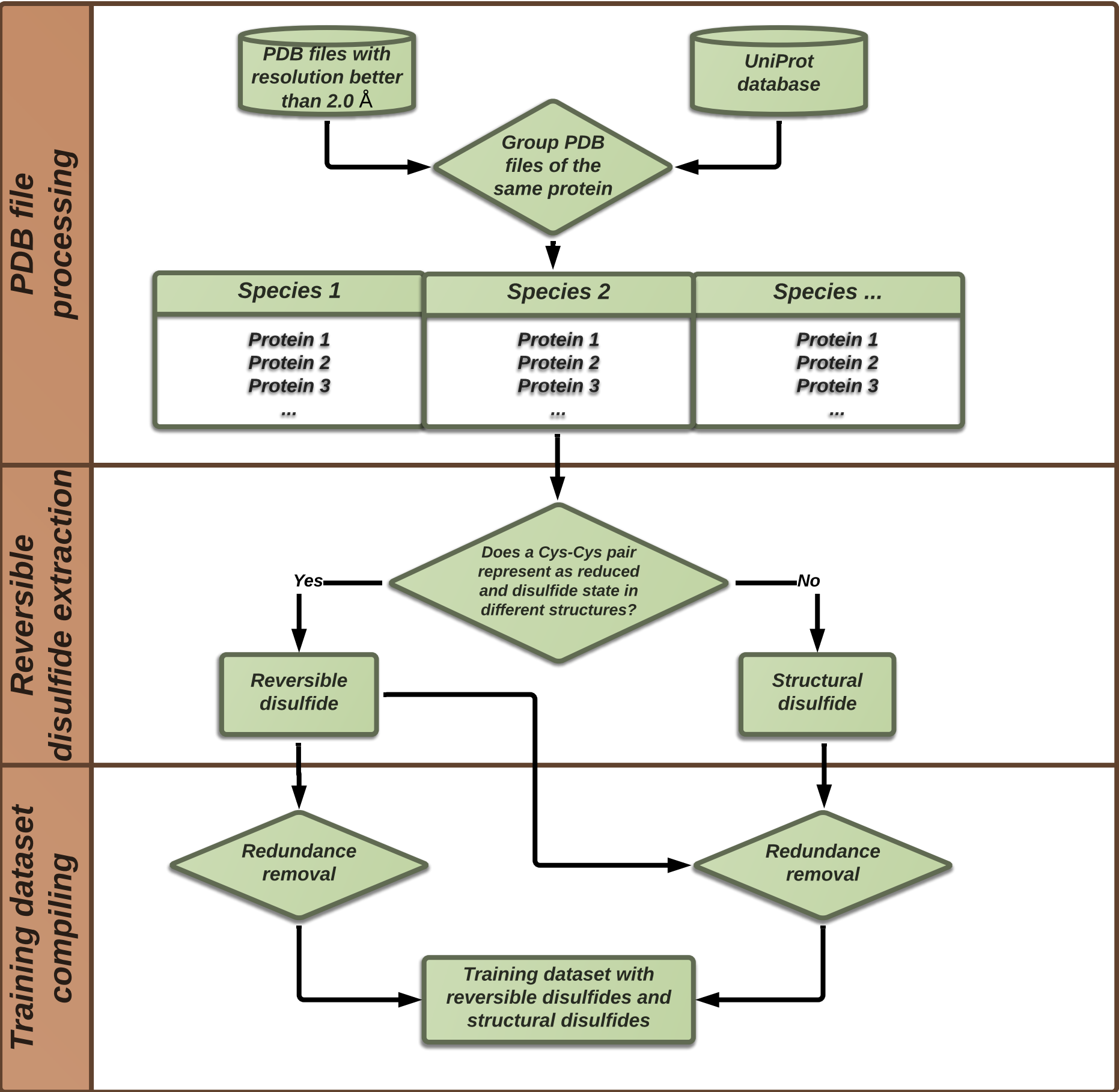

Supplement: Supplementary file 1 — Workflow for the generation of RevSS_PDB dataset by parsing protein structures from PDB database (PDF). RevSS_PDB dataset was generated from X-ray crystal structures of resolution better than 2.0 Å from PDB database. In brief, PDB files from the same proteins of the same species were first grouped together. For each group of PDB files, each Cys-Cys pair was scanned among different structures, and those represent as disulfide and reduced state in different structures were annotated as reversible disulfides. After redundancy removal using BlastClust followed by manual curation, the final RevSS_PDB dataset which contains both structural and reversible disulfides was generated. (PDF 78 kb) [file 12864_2017_3668_MOESM1_ESM.pdf]

a

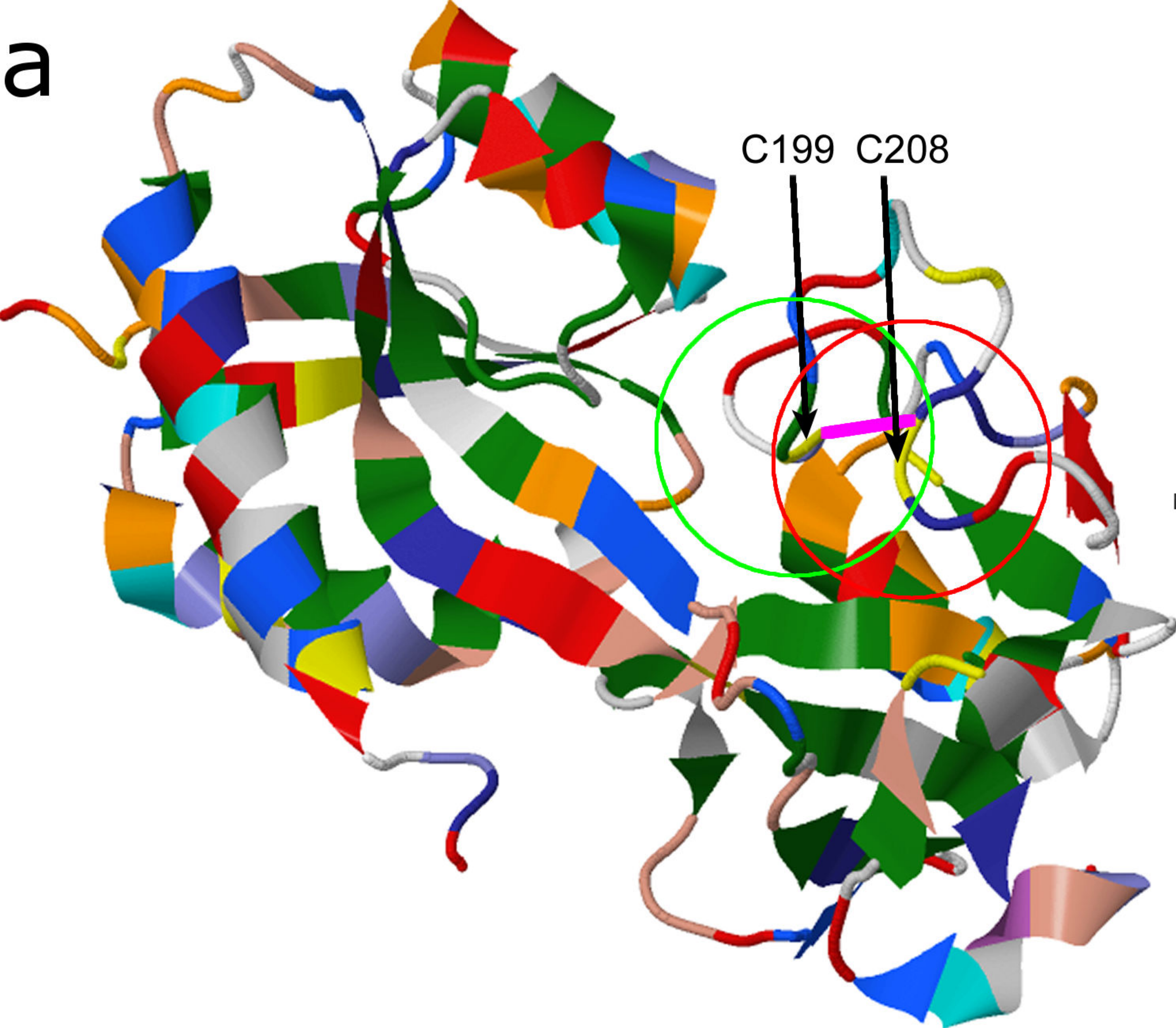

b

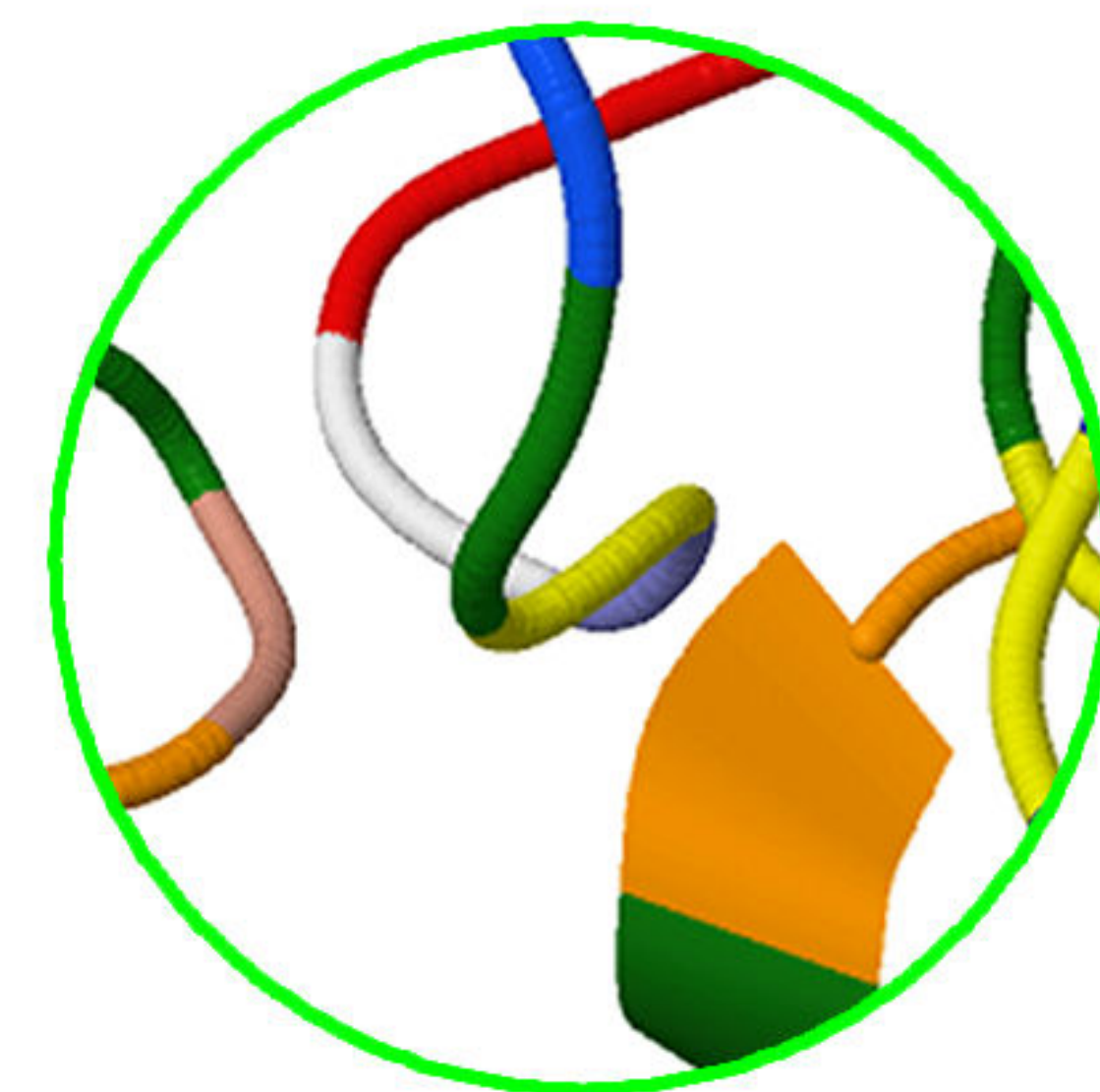

>116A A C199  
AMLE $\bar{D}$ GH $\bar{C}$ LRDQFCFELR

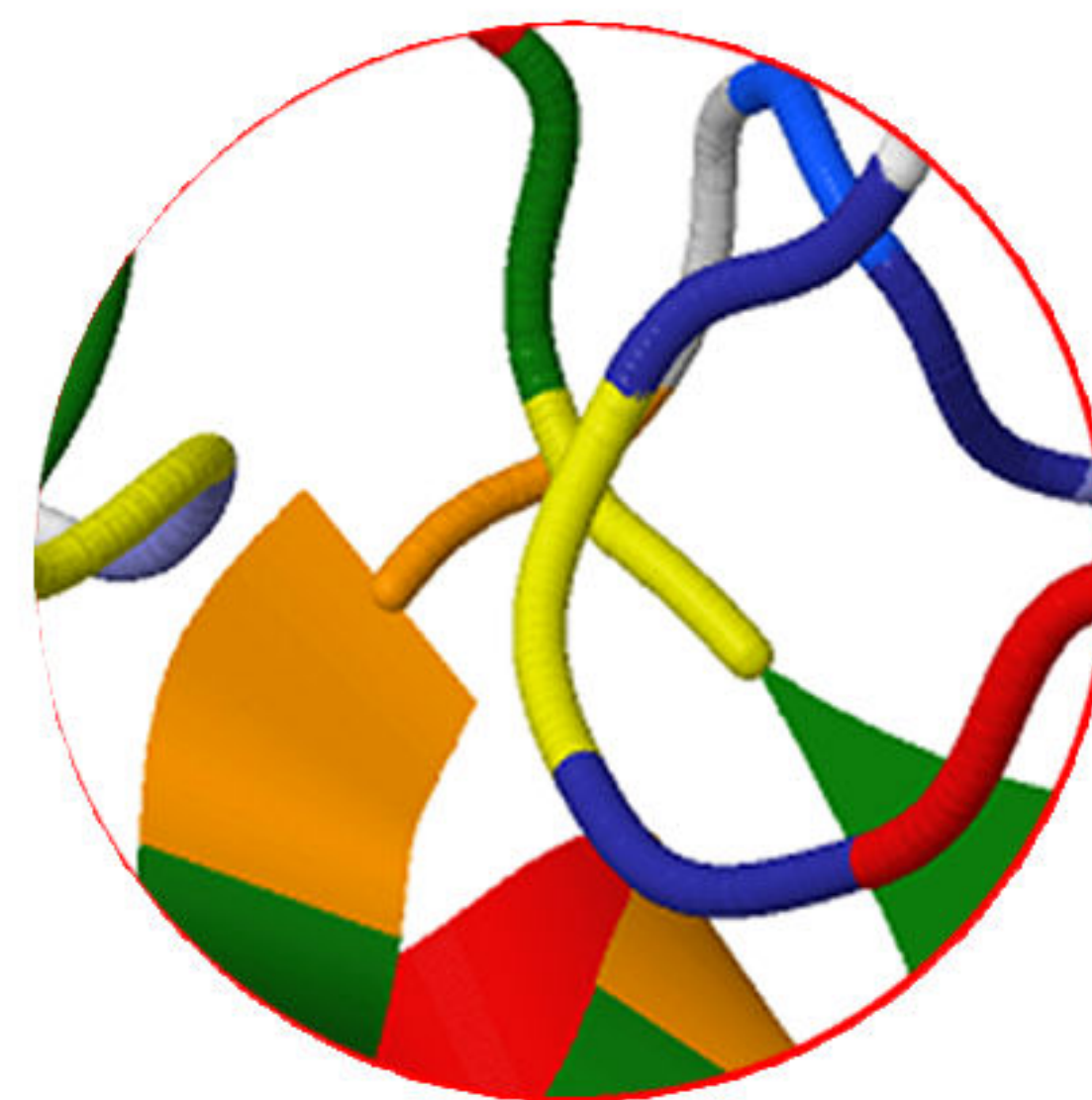

>116A A C208  
LLMLE $\bar{H}$  $\bar{C}$ LRDQAMGFCFEAAE

Supplement: Supplementary file 4 — Extraction of structural signatures surrounding disulfide in the protein structure (PDF). (a) The three dimensional structure of OxyR (PDB_ID is 1L6A) which is a transcription factor important for oxidative stress response in bacteria. The disulfide bond formed between C199 and C208 is shown as a pink bar. (b) For each Cys, the surrounding segments (within 10Å to disulfide-bonded Cys) are extracted and then combined into the so-called “structural signatures” according to their primary sequence, respectively. The signatures from the two disulfide-bonded Cys are then merged together for analysis. (PDF 374 kb) [file 12864_2017_3668_MOESM4_ESM.pdf]

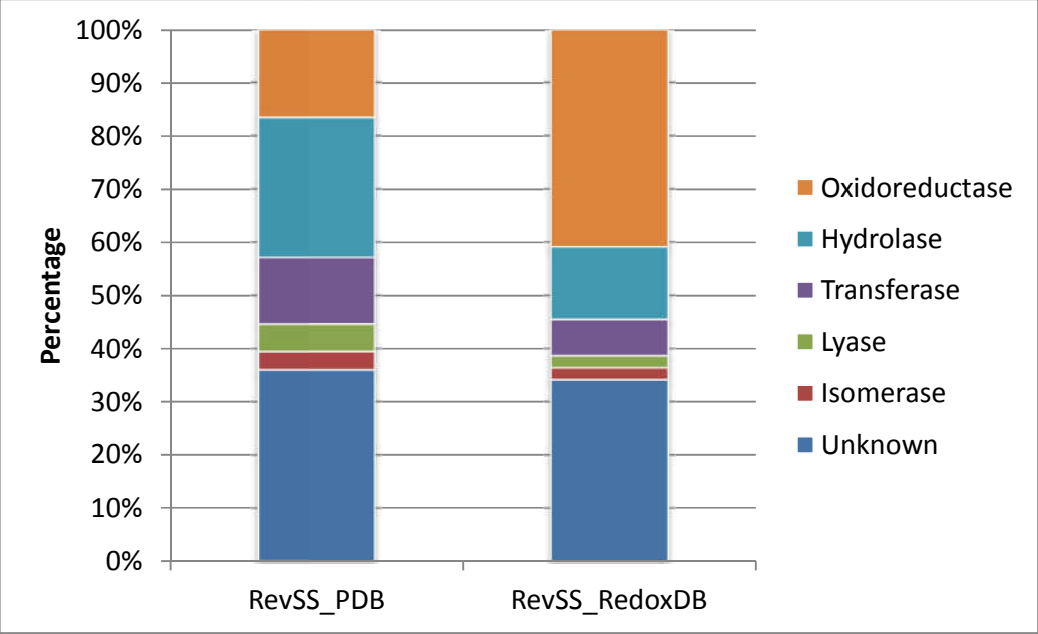

Supplement: Supplementary file 6 — Enzyme classification of reversible disulfide containing proteins in the training datasets (PDF). This figure was modified from the “Enzyme Classification” result obtained from PDB database. Only reprehensive proteins of less than 30% similarity to each other are used to generate this figure. Proteins not assigned to known enzyme groups were labeled as “Unknown”. (PDF 46 kb) [file 12864_2017_3668_MOESM6_ESM.pdf]

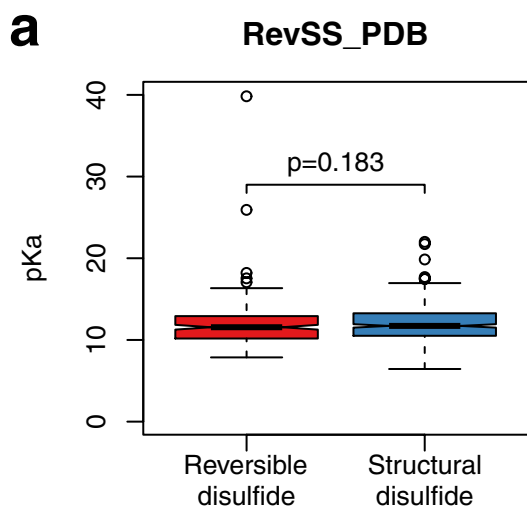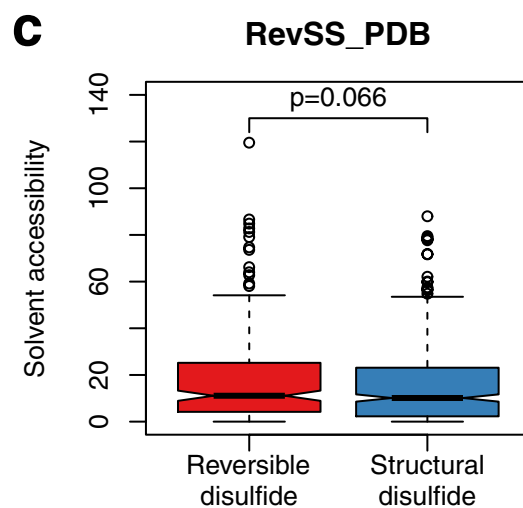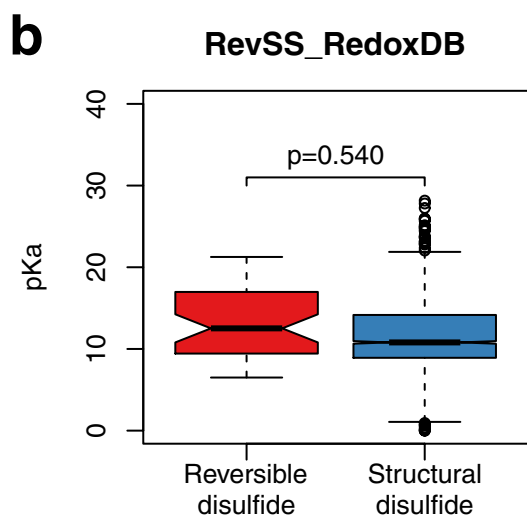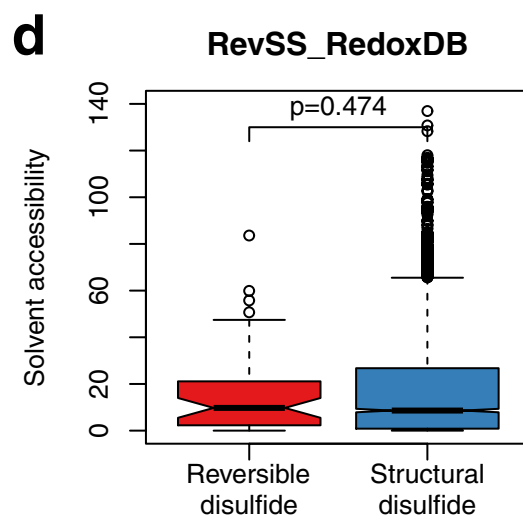

Supplement: Supplementary file 7 — Comparison of the pKa and solvent accessibility between reversible and structural disulfides (PDF). This figure shows the comparison of pKa (a,b) and solvent accessibility (c,d) between reversible and structural disulfides. Results from RevSS_PDB and RevSS_RedoxDB were both demonstrated. P-values from Two-tailed Student's t-test were indicated. (PDF 136 kb) [file 12864_2017_3668_MOESM7_ESM.pdf]

### RevSS\_PDB

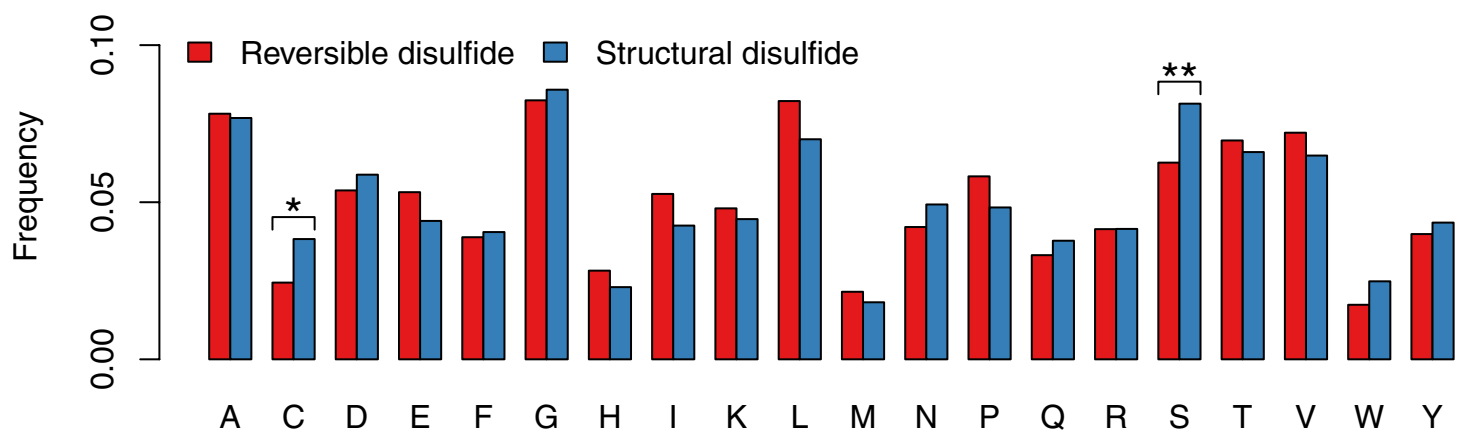

### RevSS\_RedoxDB

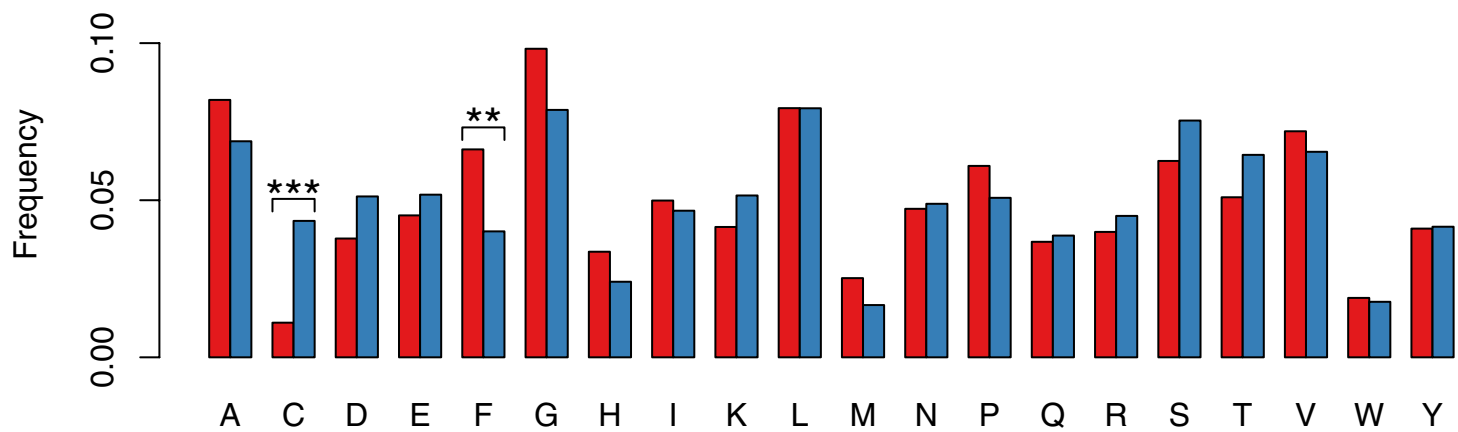

Supplement: Supplementary file 8 — Amino acid composition of the structural signatures for reversible and structural disulfides (PDF). x-axis denotes amino acid types, and y-axis gives the fraction of each amino acid in the structural signatures. Statistical significance was determined by Two-tailed Student's t-test, and Bonferroni-adjusted p-values were denoted by * when p < 0.05, ** when p < 0.01, and *** when p < 0.001. (PDF 101 kb) [file 12864_2017_3668_MOESM8_ESM.pdf]

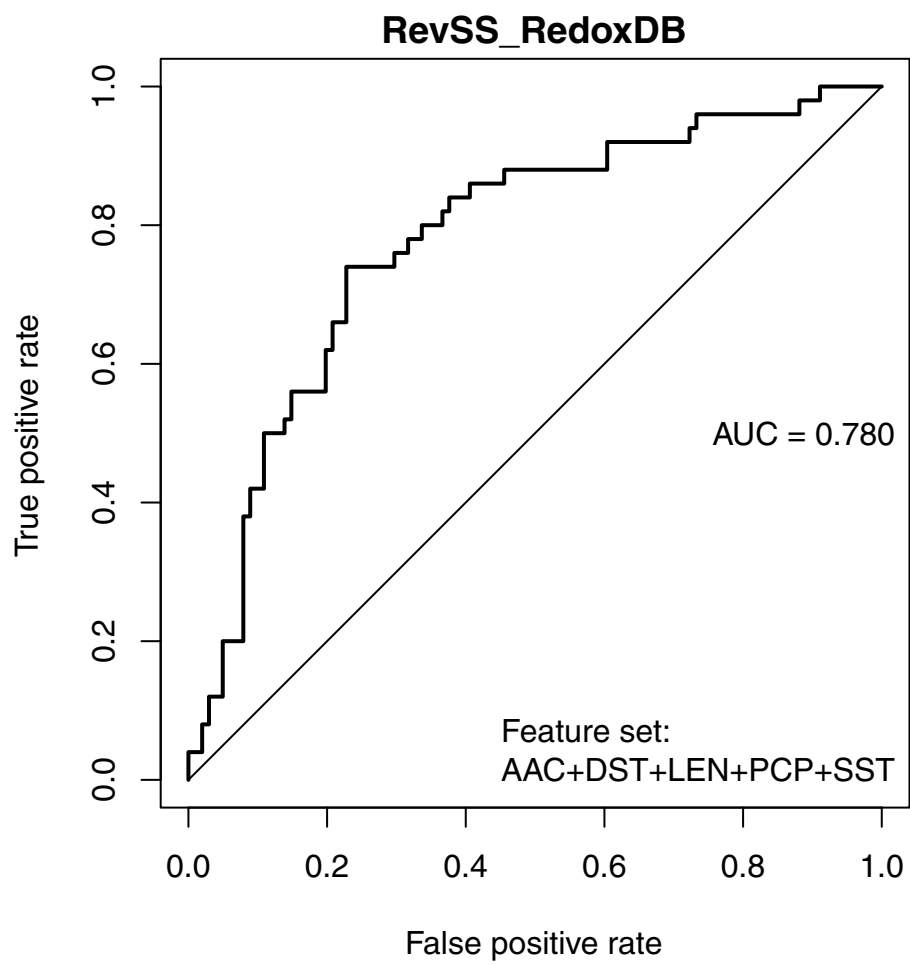

Supplement: Supplementary file 9 — ROC curve based on 10-fold cross-validation for RevSS_RedoxDB dataset (PDF). The AUC values and feature sets were indicated. (PDF 77 kb) [file 12864_2017_3668_MOESM9_ESM.pdf]

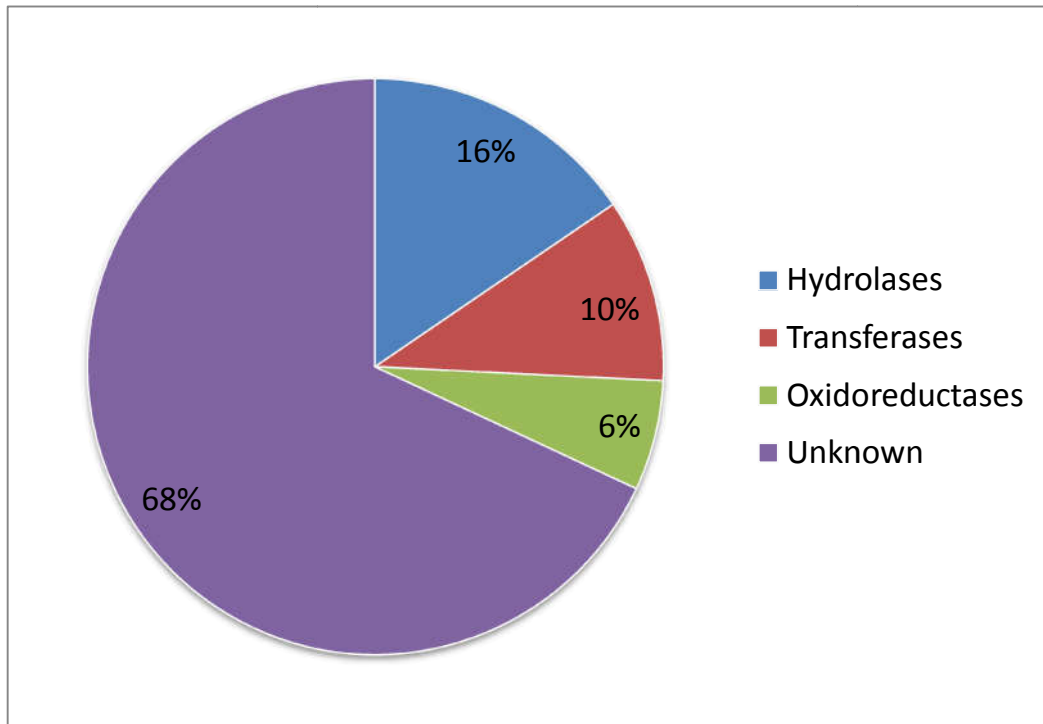

Supplement: Supplementary file 11 — Enzyme classification of proteins with predicted reversible disulfides in human (PDF). This figure was modified from the “Enzyme Classification” result obtained from PDB database. Only reprehensive proteins of less than 30% similarity to each other, and of less than 30% similarity to any of the RevssPDB sequences were used for generating this figure. Proteins not assigned to known enzyme groups were labeled as “Unknown”. (PDF 43 kb) [file 12864_2017_3668_MOESM11_ESM.pdf]
